# Chronic Cephalalgia-Parkinsonism Complex Revealing a Meningioma in a Young Patient: A Case Report

**DOI:** 10.7759/cureus.56144

**Published:** 2024-03-14

**Authors:** Yasmina Zakaria, Soukaina Benlamkadam, Hamidou Guillaume Turpin, Najib Kissani, Mohamed Chraa

**Affiliations:** 1 Neurology, Mohammed VI University Hospital, Marrakesh, MAR; 2 Neuroscience, Neuroscience Laboratory, Faculté de Médecine et de Pharmacie de Marrakech (FMPM), Marrakesh, MAR; 3 Neurosurgery, Mohammed VI University Hospital, Marrakesh, MAR

**Keywords:** brain imaging, improvement, meningioma, headache, parkinsonism

## Abstract

The emergence of parkinsonism in a patient with an intracranial meningioma is indeed an uncommon occurrence. Here, we detail the case of a patient experiencing parkinsonian syndrome for four years without any observable clinical improvement following medical treatment. A magnetic resonance imaging (MRI) of the brain revealed a left intracranial meningioma. The successful complete surgical removal of the tumor led to the resolution of parkinsonian syndrome. The extent of the neoplasm and the surrounding peritumoral edema could potentially exert significant pressure, thereby compromising perfusion in the basal ganglia region. This clinical case serves as an exemplar, emphasizing the criticality of identifying specific red flags that necessitate further clinical investigations in the context of parkinsonian syndrome.

## Introduction

Parkinson’s disease (PD) is primarily attributed to an idiopathic, progressive, and irreversible degeneration of dopaminergic neurons within the nigro-striatal system. It typically manifests with a resting tremor on one side, bradykinesia, rigidity, and gait disturbances in advanced stages [1,2]. Nevertheless, this manifestation can also arise as a secondary condition due to factors, such as stroke, drug overdose, carbon monoxide or manganese toxicity, and, rarely, a brain tumor. Parkinsonism resulting from intracranial tumors is uncommon, occurring in only 0.3% of supratentorial tumors [3].

We describe a compelling case involving a sizable meningioma in the anterior cranial fossa, exerting mass effect on the caudate nucleus, and resulting in secondary parkinsonism.

## Case presentation

This is the case of patient A.E., aged 48, with a family history of idiopathic PD in blood relatives. He reported having persistent, holocephalic headaches refractory to analgesics for the preceding four years. He also reported concomitant progressive slowing of movements globally but involving his right side predominantly. On clinical examination, A.E. presented right half-body parkinsonism characterized by bradykinesia and plastic rigidity with a cogwheel phenomenon. He had no resting tremors. His symptoms were refractory to dopamine therapy.

**Figure 1:**
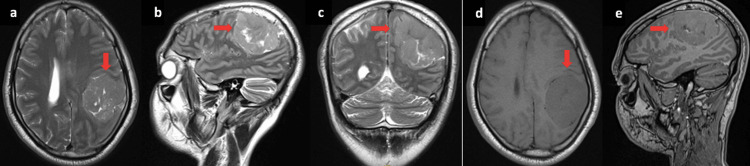
Cerebral MRI: (a) T2 axial, (b) T2 sagital, (c) T2 coronal, (d) T1 axial, (e) T1 sagital

Brain magnetic resonance imaging (MRI) revealed a left parietal tumor measuring 56×48 mm with a broad dural attachment and showing an isointense signal on T1 and T2, without perilesional vasogenic oedema, compressing the left basal ganglia, with slight enhancement after contrast injection. This tumor exerts a mass effect with midline shift and subfalcine herniation (Figure 1). The patient underwent surgery with complete excision of a single, superficial, and well-defined whitish lesion. The pathological examination of the operative specimen confirmed a Grade 1 meningotheliomatous meningioma according to the 2021 WHO classification.

The evaluation of the patient 20 days postoperatively noted a partial resolution of rigidity, with the persistence of bradykinesia.

## Discussion

Symptoms resembling idiopathic PD can be misleading in patients with slowly growing or benign brain tumors, leading to potential misdiagnosis. However, an early and accurate diagnosis can be achieved through neuroimaging studies, such as computed tomography or MRI of the brain, which can aid in reducing disease progression and enabling timely intervention. Parkinsonism can be triggered by tumors situated in various brain regions beyond the nigrostriatal pathway, including the midbrain, thalamus, and cingulate cortex, as reported in specific case studies [4,5].

Meningioma is the most frequently associated brain tumor type with the development of resting tremors, rigidity, and/or bradykinesia (seen in 70% of cases). These tumors often originate from the frontal or temporal skull base, sphenoid ridge, or frontal or temporal falx, and their size, accompanied by edema, can lead to significant compression of the basal ganglia. Due to the potential impact of the tumor on different brain segments, individuals with tumoral parkinsonism rarely exhibit exclusive parkinsonian symptoms. Parkinsonism is typically unilateral, often contralateral to the cerebral lesion, and is linked to other neurological manifestations, such as seizures, behavioral changes, or headaches, as observed in our patient [2,3]. A significant distinction between idiopathic PD and secondary parkinsonism lies in the fact that idiopathic PD usually shows a more favorable response to pharmacological dopaminergic treatment when compared to secondary parkinsonism. The parkinsonian presentation may result from direct compressive effects on the basal ganglia, particularly the nigrostriatal structures, or disruptions to frontostriatal connections.

In the described case, mechanical compression of the basal ganglia occurred without direct involvement of the basal ganglia itself. The potential for a purely incidental relationship should also be considered in patients with tumor-induced parkinsonian syndrome. Following tumor removal, the postoperative period is crucial for observing any regression of symptoms [6,7]. If no improvement occurs, it would be inappropriate to attribute the condition solely to meningioma-induced parkinsonism, although chronic compression of the basal ganglia may result in residual parkinsonian features [3,8]. Given that parkinsonian symptoms disappeared after the operation in our case, we can confidently assert a causal relationship. This case underscores the significant role of neuroimaging in patients exhibiting features suggestive of PD, especially in those with a relatively younger age at onset and the presence of red flags, such as headache or unresponsiveness to medication.

## Conclusions

The typical symptoms of parkinsonism seldom arise due to compression or infiltration by a neoplasm on the basal ganglia. In such instances, clinical manifestations are typically one-sided and are accompanied by other neurological symptoms. When dealing with cases of parkinsonian syndrome unresponsive to levodopa treatment, especially in young patients or those presenting additional neurological symptoms, like headaches, vision impairment, balance issues, or brainstem dysfunction, it is advisable to pursue more specific diagnostic investigations. This approach allows for the early identification of any underlying primary lesions, reducing progression and enabling early intervention. This, in turn, helps prevent chronic damage to the basal ganglia caused by mass effect and minimizes the risk of residual symptoms.
